# Daily sleep and physical activity from accelerometry in adults: Temporal associations and lag effects

**DOI:** 10.1016/j.sleh.2024.12.001

**Published:** 2025-01-13

**Authors:** Sarah Alismail, Calvin P. Tribby, Jiue-An Yang, Dorothy D. Sears, Noemie Letellier, Tarik Benmarhnia, Marta M. Jankowska

**Affiliations:** a Population Sciences, Beckman Research Institute, City of Hope, Duarte, California, USA; b College of Health Solutions, Arizona State University, Phoenix, Arizona, USA; c Scripps Institution of Oceanography, UC San Diego, La Jolla, California, USA; d Univ Rennes, Inserm, EHESP, Irset (Institut de recherche en santé, environnement et travail), UMR_S 1085, Rennes, France

**Keywords:** Sleep quality, Distributed lag effect, Cumulative physical activity, Objective health behavior measurement, Time effects

## Abstract

**Objectives:**

Insufficient sleep is linked to various health issues, while physical activity is a protective measure against chronic diseases. Despite the importance of sleep and physical activity for supporting public health, there remains scant research investigating daily and cumulative associations between objectively measured physical activity and sleep. Understanding the associations of physical activity and sleep behaviors over multiple days may inform the efficacy of interventions to synergistically support both behaviors.

**Method:**

Data were from the Community of Mine study (N = 367 with complete data). Participants wore ActiGraph GT3X+ accelerometers on their wrist and hip for 14 days. Sleep was defined as total sleep time (h/night), wakefulness after sleep onset (min), and sleep efficiency (%). Moderate to vigorous physical activity was defined as ≥760 counts per minute. Mixed-effects linear models with distributed lag effects, adjusted for age, Hispanic/Latino ethnicity, body mass index, education, smoking, and residence type, investigated the effect of sleep on prospective moderate to vigorous physical activity (and moderate to vigorous physical activity on prospective sleep): on the same or previous day, 2-day lag, and 3-day lag.

**Results:**

An increase in same day, 2-day lag, and 3-day lag moderate to vigorous physical activity was associated with decreased total sleep time. Moderate to vigorous physical activity was not associated with sleep efficiency or wakefulness after sleep onset. An increase in same day and 3-day lag of total sleep time was associated with decreased moderate to vigorous physical activity. An increase in 3-day lag sleep efficiency was associated with decreased moderate to vigorous physical activity. wakefulness after sleep onset was not associated with moderate to vigorous physical activity.

**Conclusions:**

These insights contribute to understanding the dynamic interplay between moderate to vigorous physical activity and sleep in adults, highlighting same day and cumulative associations.

## Introduction

Both physical activity (PA) and sleep are crucial health behaviors in achieving and maintaining good health,^1^ with recommended guidelines of 7–9 hours of sleep per night and at least 150 minutes of moderate-to-vigorous physical activity (MVPA) per week for adults in the US.^2,3^ Nevertheless, a considerable proportion of the population fails to meet these recommendations, with nearly one-third obtaining ≤6 hours of sleep^4^ and around 57% not adhering to PA guidelines.^5^ Insufficient sleep and limited PA are health behaviors with well-documented detrimental short and long-term consequences on public health, including an estimated 5.3 million excess deaths annually attributed to physical inactivity,^6^ and a spectrum of health issues (e.g., cardiovascular disease) associated with insufficient sleep.^7^

Observational and experimental evidence supports that PA benefits sleep,^8–11^ for example PA is associated with beneficial effects on total sleep time (TST) and sleep efficiency (SE) that are acute (usually same day) or regular (at least 1 week of PA).^9^ Research also suggests that insufficient sleep may also reduce PA levels,^12,13^ implying a dynamic interplay.^14,15^ On one hand, biological factors, such as homeostatic sleep pressure and circadian rhythms play a role in stable sleep patterns,^16^ and insufficient sleep may decrease physical performance and promote fatigue during PA^13^ due to elevated cortisol levels, reduced growth hormone and prolactin concentrations, as well inflammation markers’ activation.^17,18^ On the other hand, PA can impact sleep through mechanisms like increased body and central nervous system temperature,^19^ increased productions of adenosine (a sleep-promoting neurotransmitter),^19^ and reduction of anxiety and stress.^20^

Given the crucial role of PA and sleep in independently promoting health, understanding their dynamic and joint relationships in the daily lives of adults is essential to improve mental health and reduce chronic health conditions, such as cardiovascular disease, overweight and obesity, and type 2 diabetes.^21–23^ However, a notable knowledge gap exists concerning the lag effects in this dynamic relationship. Despite the associations between sleep and PA observed in various populations, including older adults,^14^ children,^24^ and individuals with chronic pain,^25^ a gap exists concerning lag effects in this relationship using objective, device measured data. Most studies have focused on daily associations,^26,27^ neglecting cumulative lag effects. However, lag effects are important to assess as inadequate sleep on one night may take up to 4 days to recover from.^28^ If lag effects are considerable, they may be important to consider in intervention or health behavior change efforts. This study examined person-level day-to-day variations in actigraphy-assessed sleep parameters and accelerometer-derived PA. The first hypothesis, based on previous literature that found positive associations between MVPA and sleep, was that MVPA on 2 and 3 lagged days would be associated with better sleep outcomes compared to the same day MVPA. That is, more consistent MVPA would be associated with better sleep. The second hypothesis, also based on previous literature that found positive associations between sleep and next day MVPA, was that better sleep outcomes on multiple lagged days would be associated with higher MVPA, compared to previous night’s sleep. The goal is to comprehensively understand the temporal dynamics of this relationship, including cumulative lag effects, essential for developing targeted behavioral interventions to improve health outcomes.

## Participants and methods

### Participants and settings

Community of Mine (CoM) is an observational study carried out between 2014 and 2017 in San Diego County, California, USA. The study collected behavioral, clinical, and biomarker outcomes related to cancer risk with the main study aim of advancing cancer risk exposure assessment; full details have been published elsewhere.^29^ Briefly, 602 adult participants (ages 35 to 80, 42% identified as Hispanic/Latino, and 56% were female) were selected from a stratified random sample from urban and suburban census block groups to maximize variability in built environment walkability. Inclusion criteria were participants must have lived in a census block group selected for the study for at least 6 months, be able to walk without human assistance, be able to travel to a study site, have a phone, be able to read and write fluently in English or Spanish, be able to give informed consent and comply with the protocol, and be willing and able to complete all assessments.^29^ Exclusion criteria were being pregnant or nursing, having a mental state that would preclude complete understanding of the protocol or compliance, having a medical condition that would affect PA or diet, or having a medical condition known to increase inflammation biomarkers.^29^

All participants provided written informed consent prior to participating in the study. Following consent, participants were mailed study devices (a hip-worn accelerometer and a wrist-worn accelerometer) and questionnaires through the mail. Approximately 1 week later, participants underwent an in-person clinic visit, during which anthropometric, vitals, and medication data were gathered. At this time, participants were given a new set of study devices (both accelerometers, due to memory limitations) and an additional questionnaire to complete for the following week. After a total of 14 days, participants returned the devices and completed questionnaires via mail. Demographic characteristics (e.g., age, sex) were obtained through completion of a self-reported survey. The full study protocol and eligibility criteria are described elsewhere.^29^ The study was approved by the University of California, San Diego’s Institutional Review Board and conducted in accordance with the Declaration of Helsinki (Protocol # 140510).

### Accelerometer data: Sleep parameters

Sleep behavior data were measured by nondominant wrist-worn accelerometers (ActiGraph GT3X+; ActiGraph, LLC; Pensacola, FL) for 24 hours per day.^29^ Additionally, participants were asked to complete a sleep journal with time to sleep and time to wake each night. Nonwear time was removed after being classified by 60 minutes of consecutive zeros in vector magnitude. Accelerometer wear was visually reviewed and coded by research staff to identify major sleep periods, periods of nonwear, and sleep journal-data discrepancies. Valid sleep nights had both a sleep journal entry and accelerometer data, with details previously published.^30^ These data were used to estimate TST (duration of hours spent asleep per night), wake after sleep onset (WASO, period of wakefulness experienced after initially falling asleep, minutes per night) and SE (percentage of time asleep during each sleep period). Sleep parameters were determined using the Cole-Kripke algorithm.^31^ These parameters served as primary predictors or outcomes in the analytical models. Wrist accelerometry generally exhibits high sensitivity (> 90%) and moderate specificity (30%-40%) in measuring TST compared to polysomnography.^32^

### Accelerometer data: PA monitoring

Participants wore ActiGraph GT3X+ accelerometers (ActiGraph, LLC; Pensacola, FL) on their hip for 14 days during waking hours, taking them off for sleep and during showering or swimming. Those who did not adhere to the protocol of wearing the accelerometer for a minimum of 7 days with at least 10 hours of daily valid wear time were requested to wear the devices again. Nonwear time (not valid) was excluded using the validated Choi algorithm^33^ in Actilife 6, which defines invalid data as 90 consecutive minutes of zero counts within a 2-minute tolerance and a 30-minute small window for detecting artificial movement. The count unit is a measure that quantifies acceleration within a time interval.^34^ MVPA was calculated using Matthews cut point of ≥760 counts/min.^35^ This cut point was selected because it was developed to capture both ambulatory and lifestyle activities and therefore better estimated overall PA.^36^

### Covariates

Covariates included age (years), sex (female, male), Hispanic/Latino ethnicity (yes/no), as prior work shows sleep and MVPA variations among these demographic groups.^37^ Other covariates associated with PA and sleep included current smoking status (yes/no),^38^ residence type (single family home, multifamily/apartment, others),^39^ and body mass index (BMI; kg/m^2^). BMI at baseline was included as people with obesity are more likely to have shorter TST and lower PA and assuming no short-time change in BMI due to changes in PA or sleep.^40,41^ BMI was calculated by dividing weight by height squared, based on measurements taken twice during the clinic visit. Height and body weight were measured barefoot with a stadiometer and a bariatric digital scale, respectively.

### Statistical analysis

Sample characteristics were presented using median and interquartile range (IQR) for continuous variables, and frequencies with percentages for categorical variables. To examine associations between daytime PA and nighttime sleep, we aligned PA and sleep on the same day. To explore whether nighttime sleep predicts daytime PA on the following day, we temporally aligned MVPA with sleep on the previous night.

Statistical analyses used R, version 4.2.2 (R Foundation for Statistical Computing, Vienna, Austria). After temporally aligning the data, linear mixed-effects models (with days nested within participants) were employed to examine the effect of daytime PA on nighttime sleep, and vice-versa, using the *lme4* package (summary of models in Table 1; graphical representation in Supplementary Figs. S1 and S2, respectively).^42^ The models’ covariance matrix structure was specified as unstructured. These models included random intercepts to account for repeated measures within each participant and random slope for the MVPA or sleep exposures to allow for heterogeneous effects (i.e., slopes) across participants. We examined the lag effect of each of the sleep parameters on MVPA and vice versa using linear mixed-effect models adjusted for age, sex, Hispanic/Latino ethnicity, BMI, smoking, and residence type. Statistical inferences were based on 95% confidence intervals (95% CI), using 5000 bootstrap samples of coefficient estimates. Bootstrapped CIs were used to facilitate the combination of multiple coefficients from the lagged exposures. We restricted our analyses to the participants with complete data for at least 5 consecutive days, with ≥10 hours of daily accelerometer wear time, so that the sample size between the six models was consistent. Due to incomplete study data (i.e., < 5 consecutive days), 235 participants were excluded from the study sample while 367 participants had a minimum of 5 consecutive days and nights of objectively measured MVPA and sleep parameters.

## Results

### Study sample

Out of the 367 included participants, 196 were female (53.4%), 152 self-identified as Hispanic/Latino (41.4%), 223 were married/partnered (60.8%), and 305 had at least some college, associate’s, bachelor’s, or graduate degree (83.1%) (Table 2). The median age was 59.5 years (IQR: 18.6 years). The median BMI was 27.6 (IQR: 6.8). Only 29 were current smokers (7.9%) and 290 reported living in single-family homes (79%). For comparison to excluded participants (due to missing data), Table 2 also contains their summary statistics; there are no significant differences in characteristics between the two subsamples.

Table 3 summarizes descriptive statistics of the actigraphy-estimated sleep parameters and accelerometer-derived MVPA. Participants engaged in a median of 103.4 daily minutes of MVPA, with median values of 6.8 hours for TST, 52.8 minutes for WASO, and 87.3% for SE per night.

### Daytime PA predicting nighttime sleep parameters

There was a significant negative association between MVPA and TST, where an increase in MVPA was associated with reduced TST (Table 4). Specifically, for every additional standard deviation (SD) in same day MVPA, TST was estimated to decrease by 0.20 SD (95% CI: − 0.37, − 0.03). Additionally, there were significant cumulative effects for 2-day and 3-day MVPA on TST. For each additional SD in MVPA, there was an estimated reduction of 0.26 (95% CI: − 0.39, − 0.14) and 0.19 (95% CI: − 0.30, − 0.09) SD in TST, for 2-day and 3-day lag models, respectively (Table 4). However, no significant same day effects were observed for MVPA, and there were no significant cumulative lag effects observed for 2-day or 3-day periods on WASO and SE (see Table 4).

### Nighttime sleep parameters predicting daytime PA

Our investigation into the associations between nighttime sleep parameters and daytime MVPA encompassed both previous day and cumulative effects, with a focus on previous day exposure, 2-day lag, and 3-day lag (Table 5). For the previous day effect, an inverse association was observed between TST and MVPA, with a coefficient of − 0.13 (95% CI: − 0.23, − 0.03). This implies that each additional SD in TST on the preceding day is associated with a reduction of 0.13 SD in MVPA.

While we did not observe any associations for the cumulative 2-day lag effect of TST on MVPA (coefficient: − 0.06, 95% CI: − 0.13, 0.01), extending the exposure window to a 3-day lag revealed a significant negative association (coefficient: − 0.06, 95% CI: − 0.12, − 0.01) (Table 5). This indicates that each additional SD in TST over the past 3 days is associated with a decrease of 0.06 SD in MVPA. In contrast, WASO did not show a significant association with MVPA across the previous day, 2-day, or 3-day lag models (Table 5). For SE, a significant negative association with MVPA was observed in the cumulative 3-day lag model (coefficient: − 0.53, 95% CI: − 1.07, − 0.01).

## Discussion

This study explored the dynamic interplay of MVPA and sleep parameters with both same day or previous day and cumulative lag effects within an ethnically diverse sample of adults aged between 35 and 80 years. This investigation revealed three key findings related to the interplay between PA and sleep parameters. First, negative associations between daytime MVPA and prospective nighttime TST were observed within a single day and cumulative lag effects (up to 3 days). Interestingly, our findings indicate a dynamic interplay between sleep and PA for TST, suggesting that TST acts as both a consequence of MVPA and a predictor of MVPA. Second, additional TST on the preceding night was linked to reduced MVPA; cumulative 3-day TST was also associated with decreased MVPA. Finally, a negative association was found in cumulative 3-day lag for SE linked to reduced MVPA. Collectively, these findings contribute valuable insights into the dynamic interplay between MVPA and sleep parameters, shedding light on the short-term and cumulative effects within the studied sample of adults.

Despite the anticipated positive impact of PA, especially MVPA, on sleep, our findings reveal a surprising negative association between daytime MVPA and subsequent nighttime TST with no association observed with WASO or SE. Single-day lag model showed that more time spent in MVPA in a day was associated with shorter TST that night. Similarly, cumulative effects for 2-day and 3-day MVPA suggested a reduction in TST. These unexpected results align with findings from previous studies on older women^43^ and children,^44^ where more daytime PA is linked to reduced nighttime sleep (shorter TST), challenging the presumed positive impact of MVPA on TST. However, comparing results among studies presents challenges due to variations in studied variables, definition of MVPA, and study designs. The implications of mixed results from this study and other studies for chronic disease prevention and interventions are that group-level results are likely more consistent and useful for public health practitioners than population-level results.^9,21^

Our findings may imply that individuals with busier daytime schedules, including time set aside for PA, may experience less nighttime sleep. Furthermore, there is a cumulative and lagged effect of MVPA on nighttime sleep. However, the interpretation of these results needs to be contextualized by the sample characteristics. For example, this sample was highly educated and lived predominantly in single family homes and it is known that occupation is related to sleep duration.^45^ Other unmeasured factors may contribute to the observed associations between MVAP and sleep, and sleep and MVPA. For example, the number and ages of children may be an unmeasured confounder, as parents of young children may exercise more than adults without children and yet parents also sleep less, with women losing more sleep than men.^46,47^ Further research is needed to explore the underlying mechanisms and potential moderating factors contributing to this negative association, including the role of sedentary time.^48^ For example, reducing sedentary time in addition to increasing PA may be more beneficial for sleep than increasing PA alone.^48^ These insights will be valuable for developing interventions to optimize both PA and sleep in adults. In addition, while exercise interventions and acute exercise have demonstrated positive effects on sleep in some studies,^49–51^ not all^8,52^ support the hypothesis that daily PA fluctuations are positively associated with sleep parameters.

We also examined the predicting role of each of the objectively measured sleep parameters on MVPA. Daily WASO and SE were not associated with MVPA. Variations in these individual measures of sleep quality were not associated with MVPA in this study, but an index of overall sleep health, composed of a set of measures, may better explain sleep quality and MVPA.^22^ There was a negative association between daily TST and MVPA on the subsequent day. This unexpected finding contradicts our initial expectations, revealing that participants who slept longer engaged in fewer minutes of MVPA the following day. This result aligns with previous epidemiological studies linking excessive sleep to lower PA.^53–55^ The reduced MVPA following longer nights of sleep may be attributed to delayed wake times,^56^ limiting available time for daily activities. However, in this study, the median TST was 6.8 hours, which is not considered excessive. Additional statistical techniques, such as quantile regression or person-specific models, may help to uncover how the relationship between sleep and MVPA changes (the effect size and direction) according to the amount of sleep on the prior night.^26,27^ These results suggest a potential trade-off between sleep and PA, especially when managing demanding schedules. The implications could be that certain health behaviors, including PA, might be compromised when balancing time constraints with sleep. This highlights the need for further investigations into factors such as time availability, exercise motivation,^12^ or other influences shaping this relationship.

Strengths of this study include objectively measured PA and sleep parameters, continuous monitoring of ≥5 consecutive days of PA and sleep data (addressing a common limitation in the literature),^57^ and a focus on daily and cumulative lag effect temporal associations between MVPA and sleep parameters and vice versa. Additionally, while several studies on the relationship between PA and sleep^11,22,58,59^ rely on self-reported measures prone to recall and social desirability biases, we utilized wrist-actigraphy and hip-worn accelerometry for objective measurements, mitigating these concerns. Despite a rising number of studies using accelerometers for sleep and PA assessment,^60^ none, to our knowledge, employed two distinct devices optimized for specific measures (e.g., wrist-actigraphy for sleep, hip accelerometry for MVPA) in adults.

Limitations of our study include data were collected in San Diego, which has a mild climate year-round. These results may be different for climates with more noticeable temperature changes between seasons and photoperiod changes.^61^ Additionally, we did not assess the time-of-day effects of PA; yet, experimental evidence suggests that the effects of exercise on sleep is modestly moderated by the time of day.^62^ Although sleep variation can occur in response to daily light exposure and significant changes in photoperiod (the relative proportions of light and dark) throughout the year,^63^ the wrist accelerometer light data collected do not account for the angle of gaze needed to capture light effects on circadian rhythms. Moreover, our results are derived from observational data, and while our analyses suggested a temporal relationship between daily MVPA and subsequent TST, this study did not account for individual-level optimal sleep duration or causes of inadequate sleep. Potentially, a laboratory sleep study may measure individual optimal sleep duration and vary hours of sleep to measure the dynamic interplay of PA and sleep.^28^ Finally, while we compared characteristics from the included subsample with the excluded subsample and did not find differences, these results may be affected by selection bias. That is, participants who had enough consecutive days with valid wear time may be different from participants who were excluded from these analyses.

## Conclusion

Our results, encompassing both daily and cumulative effects, indicate that MVPA consistently exhibited a negative relationship with TST. Similarly, TST consistently demonstrates a negative relationship with MVPA. Subsequent research may investigate the relative importance of SE and WASO vs. TST with PA, exploring whether sleep quality or duration holds greater significance for PA, or if both are equally crucial. These two health behaviors likely compete for time, with the time devoted to sleep potentially conflicting with time available for PA, and vice versa.^64^ This dynamic warrants a deeper exploration to understand the nuanced interplay between PA and sleep and the potential trade-offs individuals make in managing these competing demands on their time.

## Supplementary Material

Appendix

Appendix A. Supporting information

Supplementary data associated with this article can be found in the online version at doi:10.1016/j.sleh.2024.12.001.

## Figures and Tables

**Table 1:** Details of model exposures and outcomes

	Time effect modeled	Exposure	Outcome
	Models included in Table 4		
Model 1	Same day	MVPA on day 1	Sleep parameters on night 1
Model 2	2-day lag	MVPA on day 1 + day 2	Sleep parameters on night 2
Model 3	3-day lag	MVPA on day 1 + day 2 + day 3	Sleep parameters on night 3
	Models included in Table 5		
Model 4	Previous day	Sleep parameters on night 1	MVPA on day 2
Model 5	2-day lag	Sleep parameters on night 1 + night 2	MVPA on day 3
Model 6	3-day lag	Sleep parameters on night 1 + night 2 + night 3	MVPA on day 4

Abbreviation: MVPA, moderate-to-vigorous physical activity.

Sleep parameters included total sleep time (TST), wakefulness after sleep onset (WASO), and sleep efficiency (SE), each tested separately.

**Table 2:** Descriptive statistics of included and excluded participants from the Community of Mine Study

	Included subsample (N = 367)		Excluded subsample (N = 235)		*p* ^b^
Characteristics	N	n (%)	N	n (%)	
Age^a^ (y)	367	59.5 (18.6)	235	60 (15)	.8
Female	367	196 (53.4)	235	139 (59.1%)	.2
Latino/Hispanic	367	152 (41.4)	235	100 (43)	.8
Education	365		233		.6
Less than high school		34 (9.3)		26 (11.2)	
High school diploma		26 (7.1)		13 (5.6)	
Some college, associate’s, bachelor’s, or graduate degree		305 (83.1)		194 (83.3)	
Marital status	364		233		.3
Single		49 (13.4)		24 (10.3)	
Married/partnered		223 (60.8)		140 (60.1)	
Widowed/divorced/separated		92 (25.1)		69 (29.6)	
BMI^a^ (kg/m^2^)	397	27.6 (6.8)	235	28.1 (7)	.11
Smoking	363	29 (7.9)	232	16 (6.9)	.6
Residence type	364		230		.9
Single family		290 (79.0)		183 (79.6)	
Multifamily/Apartment		57 (15.5)		35 (15.2)	
Other		17 (4.6)		12 (5.2)	

Abbreviation: BMI, Body mass index.

a Median (IQR).

b Wilcoxon rank sum test; Pearson’s chi-squared test.

**Table 3:** Descriptive data for sleep and MVPA (N = 367)

Variables	Median (IQR)^b^
Sleep (actigraphy)	
Total sleep time (h)	6.8 (1.3)
WASO (min)	52.8 (34.9)
Sleep efficiency (%)^a^	87.3 (7.0)
MVPA (min)	103.4 (66.1)

Abbreviations: IQR, interquartile range; MVPA, moderate to vigorous physical activities; WASO, wakefulness after sleep onset.

Sleep/MVPA values averaged across the total number of valid days/nights.

a Percentage of minutes scored as sleep during the downtime interval.

b *P* < .05.

**Table 4:** Daytime MVPA minutes predicting nighttime sleep parameters

	Outcome: TST, h	Outcome: WASO	Outcome: SE, %
	β (95% CI)	β (95% CI)	β (95% CI)
Model 1: Same day effect of MVPA (min)	−0.20 (−0.37, −0.03)	0.03 (−0.07, 0.14)	−0.01 (−0.03, 0.01)
Model 2: Cumulative effect of MVPA (min) with 2-day lag	−0.26 (−0.39, −0.14)	0.001 (−0.06, 0.08)	−0.01 (−0.02, 0.01)
Model 3: Cumulative effect of MVPA (min) with 3-day lag	−0.19 (−0.30, −0.09)	−0.01 (−0.08, 0.07)	6.78 (−0.01, 0.01)

Abbreviations: MVPA, moderate-to-vigorous physical activity (min); SE, sleep efficiency; TST, total sleep time; WASO, wakefulness after sleep onset.

All models adjusted for age, sex, Latino/Hispanic, body mass index (BMI), smoking status, and residence type.

**Table 5:** Nighttime sleep parameters predicting daytime MVPA

	Outcome: MVPA (min)
	β (95% CI)
Model 4: Previous day effect sleep parameters	
4a: TST, h	−0.13 (−0.23, −0.03)
4b: WASO	0.05 (−0.09, 0.21)
4c: SE, %	−0.43 (−1.26, 0.47)
Model 5: Cumulative effect of sleep parameters with 2-day lag	
5a: TST, h	−0.06 (−0.13, 0.01)
5b: WASO	0.08 (−0.03, 0.20)
5c. SE, %	−0.52 (−1.12, 0.09)
Model 6: Cumulative effect of sleep parameters with 3-day lag	
6a: TST, h	−0.06 (−0.12, −0.01)
6b: WASO	0.08 (−0.03, 0.19)
6c: SE, %	−0.53 (−1.07, −0.01)

Abbreviations: MVPA, moderate-to-vigorous physical activity; SE, sleep efficiency; TST, total sleep time; WASO, wakefulness after sleep onset.

All models adjusted for age, sex, Latino/Hispanic, BMI, smoking status, and residence type.
