# Supplementary figures and images for: Daily sleep and physical activity from accelerometry in adults: Temporal associations and lag effects
Source: Sleep Health. Author manuscript; Available in PMC 2025 Aug 2. (PMC12317671; doi:10.1016/j.sleh.2024.12.001)

**Supplement**

Figure S1. Models in Table 4.


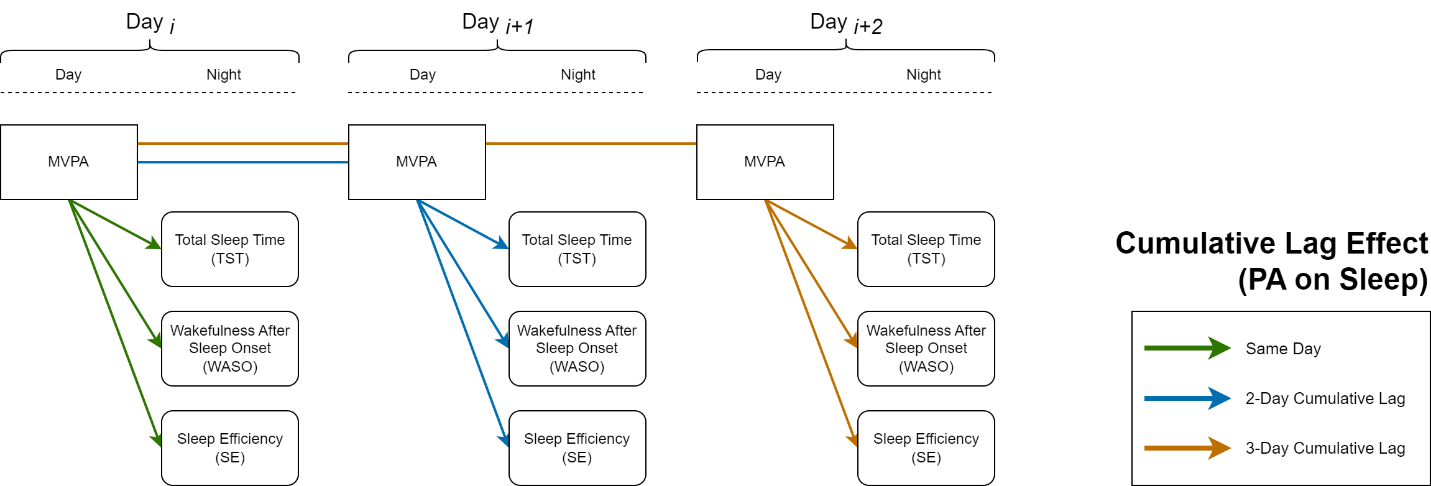


Figure S2. Models in Table 5.

**
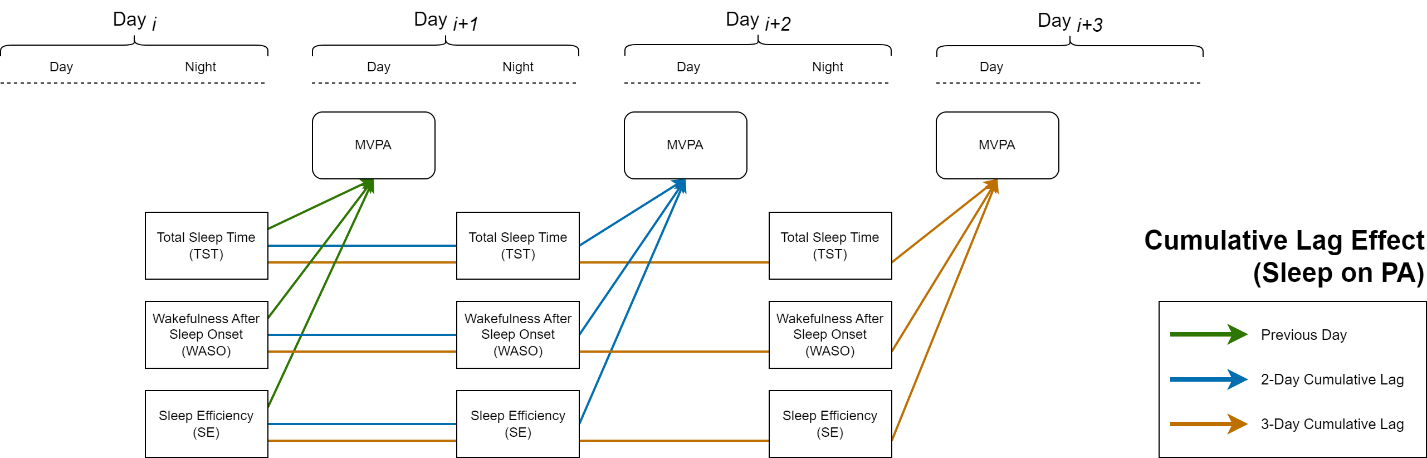
**

Supplement: Appendix [file NIHMS2098920-supplement-Appendix.docx]
